# CFRP Origami Metamaterial with Tunable Buckling Loads: A Numerical Study

**DOI:** 10.3390/ma14040917

**Published:** 2021-02-15

**Authors:** Houyao Zhu, Shouyan Chen, Teng Shen, Ruikun Wang, Jie Liu

**Affiliations:** School of Mechanical and Electric Engineering, Guangzhou University, Guangzhou 510006, China; aizhuhouyao@126.com (H.Z.); maxcsy@gzhu.edu.cn (S.C.); shent215@gzhu.edu.cn (T.S.)

**Keywords:** carbon fiber reinforced plastic, origami metamaterial, tunable buckling load

## Abstract

Origami has played an increasingly central role in designing a broad range of novel structures due to its simple concept and its lightweight and extraordinary mechanical properties. Nonetheless, most of the research focuses on mechanical responses by using homogeneous materials and limited studies involving buckling loads. In this study, we have designed a carbon fiber reinforced plastic (CFRP) origami metamaterial based on the classical Miura sheet and composite material. The finite element (FE) modelling process’s accuracy is first proved by utilizing a CFRP plate that has an analytical solution of the buckling load. Based on the validated FE modelling process, we then thoroughly study the buckling resistance ability of the proposed CFRP origami metamaterial numerically by varying the folding angle, layer order, and material properties, finding that the buckling loads can be tuned to as large as approximately 2.5 times for mode 5 by altering the folding angle from 10° to 130°. With the identical rate of increase, the shear modulus has a more significant influence on the buckling load than Young’s modulus. Outcomes reported reveal that tunable buckling loads can be achieved in two ways, i.e., origami technique and the CFRP material with fruitful design freedoms. This study provides an easy way of merely adjusting and controlling the buckling load of lightweight structures for practical engineering.

## 1. Introduction

Buckling is a failure mode that often occurs when the structure is under compressive stress. The typical characteristic of buckling is the sudden lateral displacement of the structural members, causing structural instability and leading to the critical importance of designing structures with buckling resistance ability, particularly for thin rods or thin-walled structures [1].

Generally, there are three main methods to compute the buckling loads or enhance one structure’s buckling resistance ability, i.e., analytical methods, numerical methods, and test methods. The premise of utilizing analytical methods is that the structure’s geometry is relatively simple, for example, rods, beams, and shells [2]. Test methods generally involve expensive costs, so relatively few experimental tests are conducted, which are often employed to validate analytical methods and numerical methods [3]. Various numerical methods have been proposed to deal with the buckling problems with complex geometry topology [4,5,6,7,8,9]. Although topology optimization can achieve novel designs that possess excellent buckling resistance ability, the buckling load is determined and hard to change once designed. However, a tunable mechanical property is in higher demand, which is generally realized by structural design with multiple materials [10,11] or the origami technique [12,13]. The latter is considered in this study.

Origami, originated from traditional culture, has become a powerful tool for designing novel engineering structures with various intriguing mechanical properties. An origami structure can be divided into two categories, according to whether it has an open or closed configuration. The most classical and famous origami structure is the Miura sheet, belonging to the former category, which has been extensively studied [14,15,16,17,18,19,20,21,22]. Liu, et al. studied the in-plane and out-of-plane deformation behavior of the Miura sheet using experimental and numerical methods [14]. Intriguing mechanical properties have been demonstrated for the Miura sheet using theoretical methods; the Miura sheet behaves with a positive Poisson’s ratio for in-plane deformation and a positive Poisson’s ratio for out-of-plane bending [15]. The Miura sheet was also employed as the core to enhance the out-of-plane crush dynamic property of sandwich plates [16]. Using Miura sheets to construct origami structures with a closed configuration, e.g., the Miura tube, can achieve more prosperous mechanical properties and have broader application prospects [23,24,25,26,27,28,29,30,31,32,33]. The stiffness characteristics and reconfiguration ability of a series of Miura tubes were earlier investigated by Filipov, et al. [23]. You and co-workers have proposed a new parametric modelling method for designing origami tubes; this method is validated through physical prototype testing [24]. Energy absorption characteristics of origami tubes have also been investigated numerically and experimentally [25,26,27,28]. Recently, Liu and co-workers have studied the dynamic properties, i.e., natural frequency and dynamic displacement response, of origami tubes under numerical and experimental methods [29], finding that the dynamic property can be largely tuned by altering the chief geometrical parameters. However, most of the aforementioned research is focused on the mechanical responses of origami tubes with homogeneous materials; in other words, only the potential of origami is realized. Origami tubes based on composite material can further gain more fruitful mechanical performances owing to more design freedoms in the material properties [30,31]. For instance, origami tubes’ excellent energy absorption characteristics constituted by carbon fiber reinforced plastic (CFRP) were demonstrated [32,33]. Gohari and co-authors have conducted analytical, numerical, and experimental studies to improve the failure prediction of laminated ellipsoidal woven GFRP composite domes subjected to internal pressure [34]. Wang and co-authors have proposed an integrated modelling method for FE analysis of the grinding process of long fiber reinforced ceramic matrix woven composites, the effectiveness of which was verified by experiments [35]. It is noteworthy that finite element software available for numerical simulation, e.g., Abaqus/CAE, is also an effective tool to study a composite structure’s mechanical properties [36,37]. It is known that thin-walled CFRP origami tubes are susceptible to axial compressive stress, easily resulting in buckling instability, but the study on the crucial mechanical property, buckling resistance capacity, of CFRP origami tubes is limited.

Thus, in this study, we have designed an origami metamaterial based on the Miura sheet and CFRP to fully explore the origami technique’s potential and fruitful design freedoms of composite material. Buckling loads of the proposed CFRP origami metamaterial are thoroughly investigated numerically by employing the commercial software Abaqus, with the finding that the buckling resistance capacity can be tuned to a wide range by altering the folding angle, layer order, and base material parameters. The finite element (FE) modelling process is verified using a CFRP plate with an analytical solution of the axial buckling load. The paper is organized as follows. After the Introduction, Section 2 describes the methods and materials used, including the geometric and FE modelling for the CFRP origami metamaterial, and the CFRP plate’s theoretical solution. Section 3 shows the results and discussions. The manuscript is closed with conclusions in Section 4.

## 2. Materials and Methods

### 2.1. Modelling of the CFRP Origami Metamaterial

**Figure 1:**
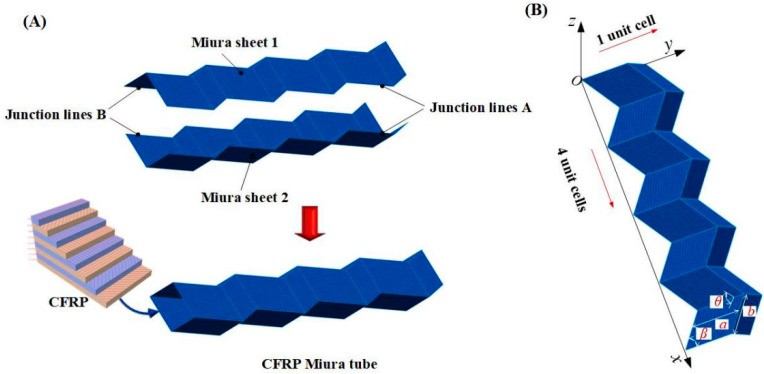
(**A**) Modelling of the CFRP Miura tube; (**B**) a typical Miura sheet with 4 by 1 unit cells.

The CFRP origami metamaterial geometrically consists of two identical Miura sheets, i.e., Miura sheet 1 and Miura sheet 2, which are symmetrical and centered on the junction surface, as shown in Figure 1A. The junction surface is determined by two junction lines, i.e., junction line A and junction line B. The Miura sheet is comprised of unit cells periodically arranged in the *x* and *y* directions. The unit cell’s geometric topology can be determined by three parameters, i.e., the length *a*, width *b*, and acute angle *β* [29]. By periodically arranging the unit cells in the *x* and *y* directions, the Miura sheet’s geometric topology is further determined by the folding angle, *θ*. These four parameters can be seen in Figure 1B, and have the following rigorous mathematical relations [29]:

(1A) $$\cos\gamma =\sin^{2}\beta \cos\theta +\cos^{2}\beta$$

(1B) $$\cos\delta =\frac{\sin^{2}\beta \cos^{2}\left(\theta /2\right)-\cos^{2}\beta }{\sin^{2}\beta \cos^{2}\left(\theta /2\right)+\cos^{2}\beta }$$

(1C) w=2bsin(γ/2)

(1D) h=acos(δ/2)

(1E) l=2asin(δ/2)

(1F) v=bsin(γ/2)

It should be noted that, in addition to these four parameters, other parameters in Equations (1A–F) can refer to reference [27]. To be brief without losing generality, we choose the Miura sheets with one unit cell in the *y* direction and *n* unit cells in the *x* direction to construct the origami metamaterial. Therefore, the designed origami tube was finally identified by five independent parameters, i.e., the length *a*, width *b*, acute angle *β*, the folding angle *θ*, and the number of the unit cells in the *x* direction, *n*. For example, Figure 1B shows a typical case when *n* is equal to 4.

In this study, we employed carbon fiber reinforced plastic (CFRP) rather than homogeneous material, e.g., metal, to constitute the origami metamaterial to further improve the design freedom, thereby emerging the CFRP origami metamaterial as depicted in the lower part of Figure 1A.

### 2.2. Numerical Simulations

**Figure 2:**
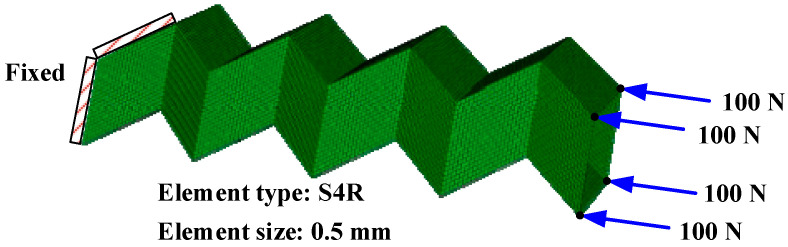
FE model of the CFRP metamaterial.

We conducted numerical simulations to identify the buckling loads of the CFRP origami metamaterial by the commercial finite element code Abaqus/Linear perturbation [38]. Two identical Miura sheets were rigidly connected at the junction surface, as described in Section 2.1. Regarding the CFRP origami metamaterial boundary conditions, all degrees of freedom on the left side were constrained. Four axial compression forces of 100 N were applied on the right end nodes; the loading was directly applied in Abaqus in the buckle step, and the type of loading was concentrated force. The whole CFRP origami metamaterial was meshed by 12,800 four-node shell elements with reduced integration (S4R) and an approximate size of 0.5 mm, as shown in Figure 2. The shell element was a four node thick shell, with reduced integration, hourglass control, and finite membrane strains. There were a total of eight layers of CFRP, symmetrically laid; the sequence of composite lamination was [45/−45/90/0], with a thickness of each layer of 0.125 mm. The material properties are listed in Table 1. *E*_1_, *E*_2_, and *E*_3_ represent the material’s elastic modulus in the main direction of elasticity 1, 2, and 3, respectively; *υ*_12_, *υ*_13_, and *υ*_23_ are the negative values of the ratio of strain in the 1 direction to strain in the 2 direction, strain in the 1 direction to strain in the 3 direction, and strain in the 2 direction to strain in the 3 direction, respectively. *G*_12_, *G*_13_, and *G*_23_ are the shear modulus in the 1–2 plane, 1–3 plane, and 2–3 planes, respectively. Material properties were set in the material editing interface, i.e., [mechanical]/[Elasticity]/[Elastic]/[Lamina]. The geometric parameters to determine the topology of the CFRP origami metamaterial are: *a* = 10 mm, *b* = 10 mm, *β* = 55°, *θ* = 130°, and *n* = 4. It should be underlined that the buckling load was obtained by multiplying the axially applied load by the Eigenvalue, and the Eigenvalues can be extracted from the numerical simulations, e.g., the buckling load of mode 1 can be calculated by multiplying the axially applied load by the Eigenvalue corresponding to mode 1; other situations are similar.

### 2.3. Theoretical Prediction of the Axial Buckling Loads for a Classical CFRP Plate

Considering the complex geometry and the complicated constitutive relationship of the proposed CFRP origami metamaterial, we employed a classical CFRP plate, which has a theoretical solution, to verify the FE modelling process in terms of the buckling load.

When the unidirectional composite is in the principal axis direction, the elastic constant is calculated as follows. The relationship between the stress and the strain is given as [39]:

(2) $$\left\{\begin{array}{l} \sigma_{1}\\ \sigma_{2}\\ \tau_{12} \end{array}\right\}=\left[\begin{array}{ccc} Q_{11} & Q_{12} & 0\\ Q_{12} & Q_{22} & 0\\ 0 & 0 & Q_{66} \end{array}\right]\left\{\begin{array}{l} \varepsilon_{1}\\ \varepsilon_{2}\\ \gamma_{12} \end{array}\right\}$$

where $Q_{11}=\frac{E_{1}}{1-\upsilon_{12}\upsilon_{21}}$, $Q_{12}=\frac{\upsilon_{21}E_{1}}{1-\upsilon_{12}\upsilon_{21}}$, $Q_{22}=\frac{E_{2}}{1-\upsilon_{12}\upsilon_{21}}$, $Q_{66}=G_{12}$, $\frac{\upsilon_{12}}{E_{1}}=\frac{\upsilon_{21}}{E_{2}}$.

When the unidirectional composite is in the principal off-axis direction, the elastic constant is calculated as follows. The stress and strain have the following relationship [39]:

(3) $$\left\{\begin{array}{l} \sigma_{x}\\ \sigma_{y}\\ \tau_{xy} \end{array}\right\}=\left[\begin{array}{ccc} {\bar{Q}}_{11} & {\bar{Q}}_{12} & {\bar{Q}}_{16}\\ {\bar{Q}}_{12} & {\bar{Q}}_{22} & {\bar{Q}}_{26}\\ {\bar{Q}}_{16} & {\bar{Q}}_{26} & {\bar{Q}}_{66} \end{array}\right]\left\{\begin{array}{l} \varepsilon_{x}\\ \varepsilon_{y}\\ \gamma_{xy} \end{array}\right\}$$

Defining the angle between the coordinate axis and the fiber direction as θ, one gets:

$${\bar{Q}}_{11}=Q_{11}{\left(\cos\theta \right)}^{4}+2\left(Q_{12}+2Q_{66}\right){\left(\cos\theta \right)}^{2}{\left(\sin\theta \right)}^{2}+Q_{22}{\left(\sin\theta \right)}^{4}\;{\bar{Q}}_{12}=\left(Q_{11}+Q_{22}-4Q_{66}\right){\left(\cos\theta \right)}^{2}{\left(\sin\theta \right)}^{2}+Q_{12}\left({\left(\sin\theta \right)}^{4}+{\left(\cos\theta \right)}^{4}\right)\;{\bar{Q}}_{22}=Q_{11}{\left(\sin\theta \right)}^{4}+2\left(Q_{12}+2Q_{66}\right){\left(\cos\theta \right)}^{2}{\left(\sin\theta \right)}^{2}+Q_{22}{\left(\cos\theta \right)}^{4}\;{\bar{Q}}_{16}=\left(Q_{11}-Q_{12}-2Q_{66}\right){\left(\cos\theta \right)}^{3}\sin\theta +\left(Q_{12}-Q_{22}+2Q_{66}\right)\cos\theta {\left(\sin\theta \right)}^{3}\;{\bar{Q}}_{26}=\left(Q_{11}-Q_{12}-2Q_{66}\right)\cos\theta {\left(\sin\theta \right)}^{3}+\left(Q_{12}-Q_{22}+2Q_{66}\right){\left(\cos\theta \right)}^{3}\sin\theta \;{\bar{Q}}_{66}=\left(Q_{11}+Q_{22}-2Q_{12}-2Q_{66}\right){\left(\cos\theta \right)}^{2}{\left(\sin\theta \right)}^{2}+Q_{66}\left[{\left(\cos\theta \right)}^{4}+{\left(\sin\theta \right)}^{4}\right]$$

Elastic properties of laminated plates can be calculated as:

(4) $$\left[\begin{array}{l} N\\ M \end{array}\right]=\left[\begin{array}{cc} A & B\\ B & D \end{array}\right]\left[\begin{array}{l} \varepsilon^{0}\\ K \end{array}\right]$$

where the internal force $N=\left[\begin{array}{l} N_{x}\\ N_{y}\\ N_{xy} \end{array}\right]$, and the internal moment $M=\left[\begin{array}{l} M_{x}\\ M_{y}\\ M_{xy} \end{array}\right]$. $\varepsilon^{0}$ is the in-plane strain. K represents the change in curvature and torsion of the middle plane before and after the deformation. A is the tensile stiffness matrix, $A=\left[\begin{array}{ccc} A_{11} & A_{12} & A_{16}\\ A_{12} & A_{22} & A_{26}\\ A_{16} & A_{26} & A_{66} \end{array}\right]$. B is the tensile and bending coupling stiffness matrix, $B=\left[\begin{array}{ccc} B_{11} & B_{12} & B_{16}\\ B_{12} & B_{22} & B_{26}\\ B_{16} & B_{26} & B_{66} \end{array}\right]$. D is the bending stiffness matrix, $D=\left[\begin{array}{ccc} D_{11} & D_{12} & D_{16}\\ D_{12} & D_{22} & D_{26}\\ D_{16} & D_{26} & D_{66} \end{array}\right]$.

Axial buckling load can be finally computed as:

(5) $$N_{x}=\frac{\pi^{2}D_{22}}{w^{2}}\left[\frac{D_{11}}{D_{12}}{\left(\frac{w}{l}\right)}^{2}\gamma^{2}+2\left(\frac{D_{12}+2D_{66}}{D_{22}}\right)+{\left(\frac{l}{w}\right)}^{2}\frac{1}{\gamma^{2}}\right]$$

where $N_{x}$ is the axial buckling load per unit length, γ is the half wave number of buckling in the direction of the plate, and l and w are the length and width of the plate, respectively.

## 3. Results

### 3.1. Validation of the FE Modelling Process for Calculating the Buckling Load

The CFRP plate was 300 mm in length and 200 mm in width, subject to a compressive load of 100 N/mm at the right side. The plate structure’s left end was fixed, and the right end was fixed with other degrees of freedom, except for the axial direction. Based on the theory presented in Section 2.3, one can get:

$$A=\left[\begin{array}{ccc} 6.1559 & 1.8995 & 0\\ 1.8995 & 6.1559 & 0\\ 0 & 0 & 2.1282 \end{array}\right]\times 10^{4}$$

$$B=\left[\begin{array}{ccc} 0 & 0 & 0\\ 0 & 0 & 0\\ 0 & 0 & 0 \end{array}\right]$$

$$D=\left[\begin{array}{ccc} 3.5940 & 2.5880 & 0.7961\\ 2.5880 & 4.6555 & 0.7961\\ 0.7961 & 0.7961 & 2.7786 \end{array}\right]\times 10^{3}$$

**Figure 3:**
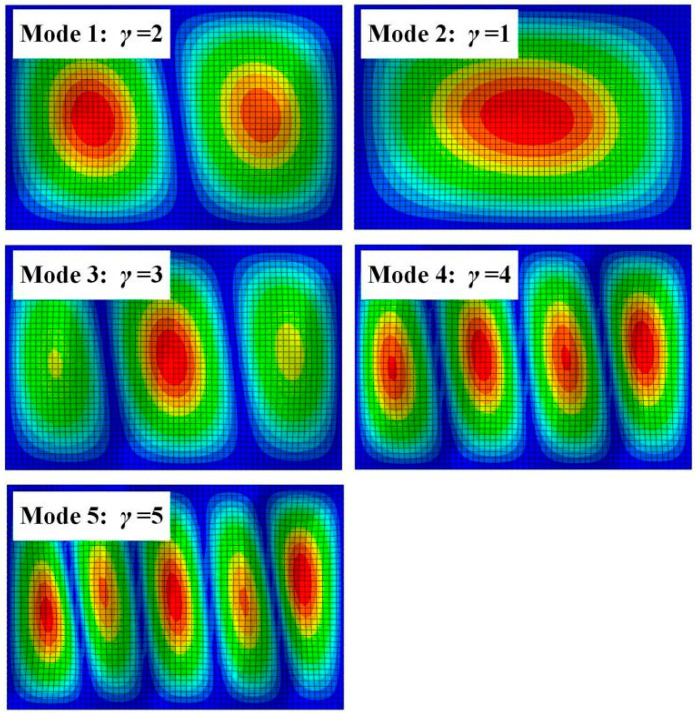
The first five buckling modes for the composite plate.

**Figure 4:**
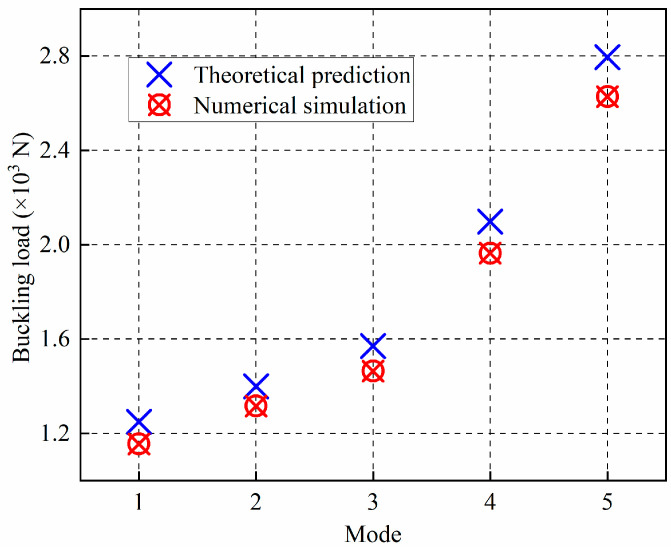
Comparison of the axial buckling loads between the theoretical prediction and numerical simulation for the composite plate.

Note that the units for the matrix ***A***, ***B***, and ***D*** are MPa·mm, MPa·mm^2^, and MPa·mm^3^, respectively. The number of half-waves can be determined from the buckling mode, as shown in Figure 3. The first five buckling modes are depicted in Figure 4, with the corresponding Eigenvalues as 5.77633 × 10^−2^, 6.57919 × 10^−2^, 7.32183 × 10^−2^, 9.82133 × 10^−2^, and 0.13141, respectively. The first five buckling loads extracted from theoretical solutions and numerical simulations are compared in Figure 4. It can be seen that the axial buckling loads obtained from the theoretical computation were 1248.4 N, 1399.6 N, 1570.8 N, 2097.4 N, and 2795.2 N, respectively, while the axial buckling loads acquired from the numerical analysis were 1155.3 N, 1315.8 N, 1464.4 N, 1964.3 N, and 2628.2 N, respectively. The numerical simulation’s relative errors compared with the theoretical computation were then calculated as −7.46%, −5.99%, −6.77%, −6.35%, and −5.97%, respectively, indicating that the FE modelling has relatively high accuracy. Therefore, we will employ the FE modelling process to predict the axial buckling loads of the proposed CFRP origami metamaterial in the succeeding paragraphs.

### 3.2. Influences of the Folding Angle

**Figure 5:**
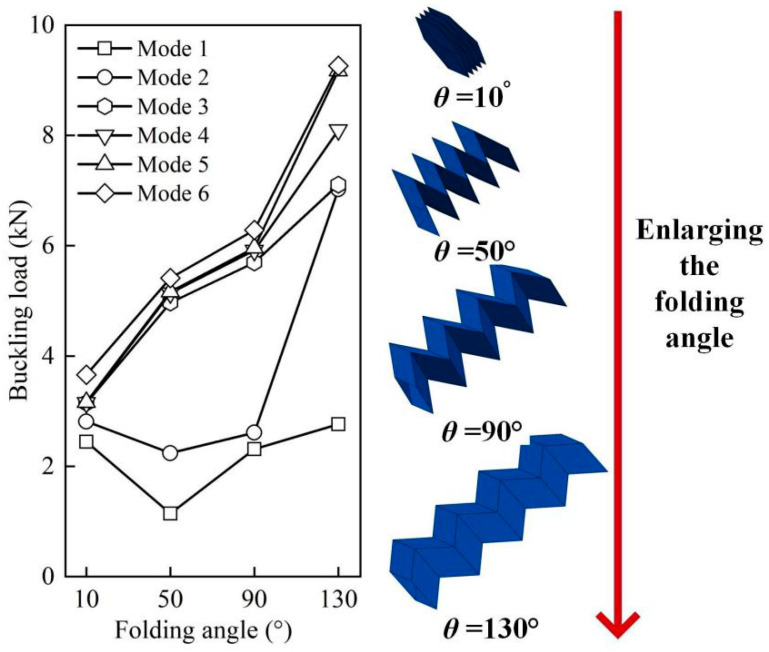
Influences of the folding angle on the buckling loads.

Figure 5 shows the influences of the folding angle on buckling loads of the CFRP origami metamaterial by varying the folding angle *θ* from 10° to 130° at an interval of 40° and with other parameters fixed. The geometric topologies of these CFRP origami metamaterials are also depicted on the right side of Figure 5. Figure 5 displays that the first six buckling loads were altered as (2.45 kN→1.14 kN→2.31 kN→2.76 kN), (2.81 kN→2.24 kN→2.61 kN→7.02 kN), (3.15 kN→4.97 kN→5.69 kN→7.11 kN), (3.15 kN→5.14 kN→5.91 kN→8.10 kN), (3.16 kN→5.17 kN→5.96 kN→9.17 kN), and (3.66 kN→5.41 kN→6.29 kN→9.26 kN), respectively, as *θ* enlarges from 10° to 130°. It can be seen that the buckling loads can be widely tuned by merely varying the folding angle, especially for high-order modes. Specifically, the larger the folding angle, the larger the buckling load for modes 3–6, while an unusual law is discovered for the first two modes, i.e., the buckling load decreased first (*θ* from 10° to 50°) and then increased (*θ* from 50° to 130°), and the buckling load changed more drastically for mode 2 than for mode 1 when *θ* exceeded 90°. It is interesting to find that, for mode 1 and mode 3, the buckling loads were approximately equal when *θ* = 10° and *θ* = 50°, respectively, enriching the design freedom of the CFRP origami metamaterial in terms of the ability to resist buckling. Moreover, it can be surprisingly found that the buckling loads can be tuned by as large as approximately 2.5 times for mode 5. Thus, the widely tuned buckling loads can be realized for the proposed origami metamaterial by altering the folding angle with the base material properties unchanged.

### 3.3. Influences of CFRP Properties

#### 3.3.1. The Layer Order

**Figure 6:**
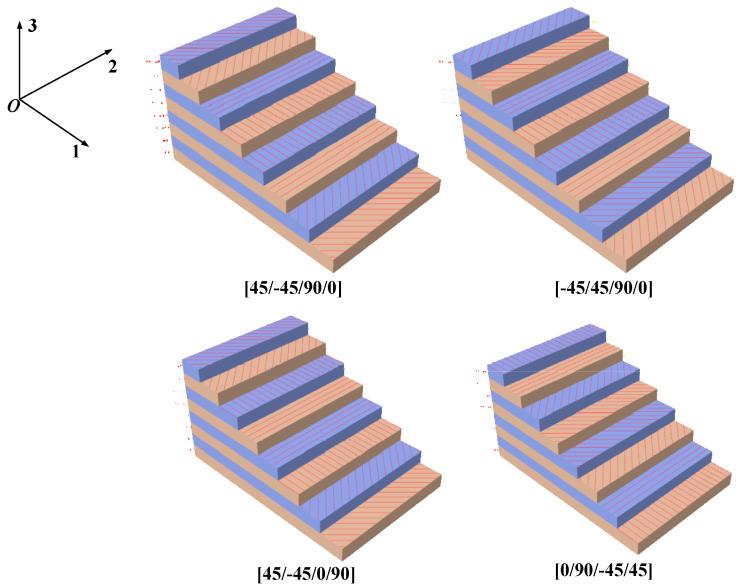
Typical layer orders for CFRP.

**Figure 7:**
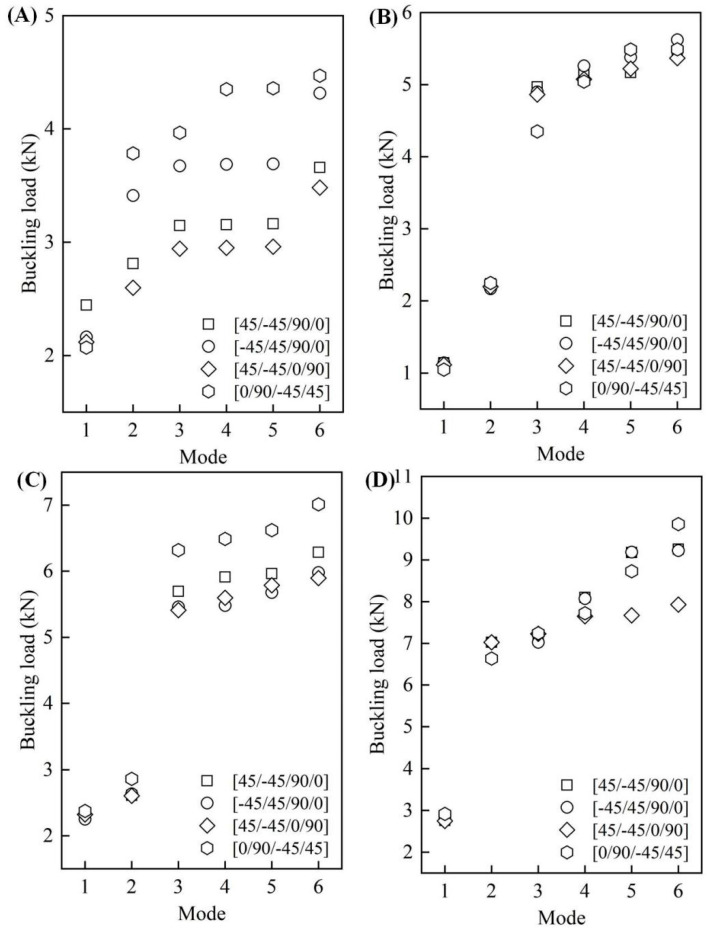
The influences of the layer order on the buckling loads. (**A**) *θ* = 10°; (**B**) *θ* = 50°; (**C**) *θ* = 90°; (**D**) *θ* = 130°.

We further investigated how the layer order of CFRP affected the buckling load of the CFRP origami metamaterial. Four specific layer orders were selected, i.e., [45/−45/90/0], [−45/45/90/0], [45/−45/0/90], and [0/90/−45/45], respectively, as shown in Figure 6. The layers were symmetrically laid, with a total of eight layers of CFRP. Each layer had a uniform thickness of 0.125 mm. Figure 7A–D shows the influences of the layer order on the buckling load when the folding angles are equal to 10°, 50°, 90°, and 130°, respectively.

When *θ* = 10°, obvious influences could be found, particularly for high-order modes. The first-order buckling loads were 2.45 kN, 2.16 kN, 2.12 kN, and 2.07 kN, respectively, for these four cases, identifying that relatively slight changes were found for the last three cases. The buckling load corresponding to modes 2–6 had an identical change in law, i.e., it became bigger first, then decreased, and then became larger; CFRP origami metamaterial with the layer order [0/90/−45/45] and layer order [45/−45/0/90] had the largest and the least buckling load, respectively. It can also be seen that, by altering the layer order, the buckling load can be tuned by as large as roughly 50% (corresponds to mode 5).

Compared with the CFRP origami metamaterial with *θ* = 10°, the buckling loads of the other three cases when the folding angles equaled 50°, 90°, and 130° were found to be relatively less sensitive to the alteration of the layer order. For example, when *θ* = 50°, all of the first-order and second-order buckling loads were about 1.1 kN and 2.2 kN, respectively. The differences in the buckling loads were almost within 5% for modes 3–6 as one turned the layer order. However, the situation changed for CFRP origami metamaterials with *θ* = 90° and 130°. For instance, when *θ* = 90°, the least and largest buckling loads were (5.4 kN, 6.3 kN), (5.5 kN, 6.5 kN), (5.7 kN, 6.6 kN), and (5.9 kN, 7.0 kN) for modes 3–6, realizing 17%, 18%, 16%, and 19% tunable properties, respectively. CFRP origami metamaterial with the folding angle of 130° had an apparent tunable buckling load performance only for high-order modes, i.e., mode 5 and mode 6. Specifically, the buckling load can be tuned from approximately 7.7 kN to 9.2 kN and from 7.9 kN to 9.9 kN, respectively, for mode 5 and mode 6 by adjusting the layer order. It was also interesting to find a significant jump in the buckling load from mode 2 to mode 3 in Figure 7C,D. This phenomenon may be caused by a large change in the buckling half-wave number in this adjacent mode. However, considering the complex geometry of the CERP origami metamaterial, this reason needs further exploration. Overall, the buckling load of the proposed CFRP origami metamaterial can be largely tuned via simply modifying the CFRP laminate layer order.

#### 3.3.2. The Material Properties

**Figure 8:**
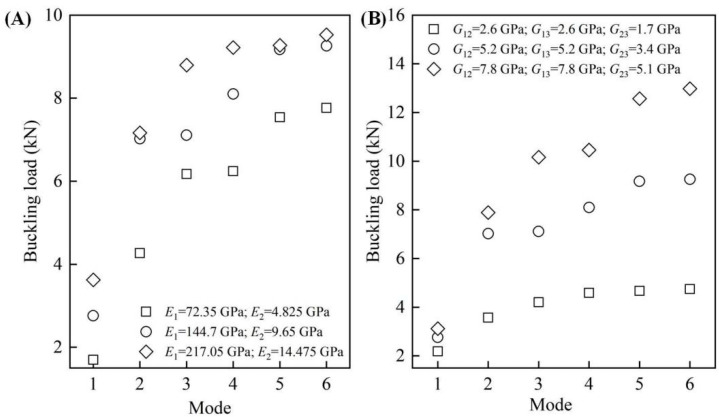
The influences of the material properties on the buckling loads. (**A**) Young’s modulus; (**B**) shear modulus.

We finally explored how the buckling load of the proposed CFRP origami metamaterial changed if the base material parameters, i.e., Young’s modulus and shear modulus, were altered. It should be underlined that the material properties (Young’s modulus and shear modulus) selection followed the following guidelines: the second group was the reference group (parameters used in the previous section); the values of the first group were half of the reference group; and the values of the third group were the first group plus the second group. Figure 8A shows the influences of Young’s modulus on the buckling loads with other material properties unchanged. Three representative cases were considered, i.e., CFRP origami metamaterial with Young’s modulus of (*E*_1_ = 72.35 GPa, *E*_2_ = 4.825 GPa), (*E*_1_ = 144.7 GPa, *E*_2_ = 9.65 GPa), and (*E*_1_ = 217.05 GPa, *E*_2_ = 14.475 GPa), respectively. It can be found that increasing the Young’s modulus values leads to the increase of the buckling load for all of the modes, revealing that Young’s modulus has a positive influence on the ability to resist buckling instability. For example, the first-order buckling load can be enlarged by roughly 112% (from 1.7 kN to 3.6 kN) as Young’s modulus tends to be larger. It is also interesting to find that there are two special cases in which the buckling loads were almost kept constant, although Young’s modulus was enlarged by 1.5 times (increase in Young’s modulus from *E*_1_ = 144.7 GPa and *E*_2_ = 9.65 GPa to *E*_1_ = 217.05 GPa and *E*_2_ = 14.475 GPa for mode 2 and mode 4, respectively). This unusual phenomenon can allow for the designing of the CFRP origami metamaterial with specific buckling loads. Figure 8B depicts the influences of the shear modulus on the buckling load, in which a similar change in the law can be found. The buckling load became larger with the shear modulus increase, except for mode 1, which showed a relatively insignificant change. Unlike Young’s modulus, no approximately identical buckling load could be discovered for the CFRP origami metamaterial when the shear modulus varied. Moreover, it can be found that the six-order buckling load can be improved by almost 174% when the shear modulus is increased from (*G*_12_ = 2.6 GPa, *G*_13_ = 2.6 GPa, *G*_23_ = 1.7 GPa) to (*G*_12_ = 7.8 GPa, *G*_13_ = 7.8 GPa, *G*_23_ = 5.1 GPa). By comparing Figure 8A,B, it can be found that, with the same increase rate, the shear modulus has a more significant impact on the buckling load than Young’s modulus. In summary, the buckling load of the proposed CFRP origami metamaterial can be easily tuned by simply changing Young’s modulus and the shear modulus of the base material.

## 4. Conclusions

This study has proposed a novel origami metamaterial with a wide-range tunable buckling load based on the Miura sheet and carbon fiber reinforced plastic (CFRP). The tunable buckling load property of the proposed CFRP origami metamaterial is thoroughly investigated using numerical simulations, whose finite element modelling process is verified by employing a CFRP plate with theoretical solutions. Results reveal that the ability to resist buckling instability for the CFRP origami metamaterial can be widely tuned through two ways, i.e., altering the folding angle by utilizing the origami merit and changing the layer order and base material properties by the fruitful design freedoms of the CFRP. Moreover, it seems that, with the same rate of increase, the shear modulus has a more significant influence on the buckling load than Young’s modulus. This research provides a solution for the design of a lightweight structure with the demand for tunable buckling resistance in actual engineering.

## Figures and Tables

**Table 1:** The material properties of the CFRP origami metamaterial.

*E*_1_	*E*_2_	*E*_3_	*υ*_12_	*υ*_13_	*υ*_23_	*G*_12_	*G*_13_	*G*_23_
144.7 GPa	9.65 GPa	9.65 GPa	0.30	0.30	0.45	5.2 GPa	5.2 GPa	3.4 GPa

## Data Availability

The data presented in this study are available on request from the corresponding author.
